# Role of Cortactin Homolog HS1 in Transendothelial Migration of Natural Killer Cells

**DOI:** 10.1371/journal.pone.0118153

**Published:** 2015-02-27

**Authors:** Suranjana Mukherjee, Joanna Kim, Olivia L. Mooren, Stefanie T. Shahan, Megan Cohan, John A. Cooper

**Affiliations:** Department of Cell Biology & Physiology, Washington University School of Medicine, St. Louis, Missouri, United States of America; Cambridge University, UNITED KINGDOM

## Abstract

Natural Killer (NK) cells perform many functions that depend on actin assembly, including adhesion, chemotaxis, lytic synapse assembly and cytolysis. HS1, the hematopoietic homolog of cortactin, binds to Arp2/3 complex and promotes actin assembly by helping to form and stabilize actin filament branches. We investigated the role of HS1 in transendothelial migration (TEM) by NK cells. Depletion of HS1 led to a decrease in the efficiency of TEM by NK cells, as measured by transwell assays with endothelial cell monolayers on porous filters. Transwell assays involve chemotaxis of NK cells across the filter, so to examine TEM more specifically, we imaged live-cell preparations and antibody-stained fixed preparations, with and without the chemoattractant SDF-1α. We found small to moderate effects of HS1 depletion on TEM, including whether the NK cells migrated via the transcellular or paracellular route. Expression of HS1 mutants indicated that phosphorylation of HS1 tyrosines at positions 222, 378 and 397 was required for rescue in the transwell assay, but HS1 mutations affecting interaction with Arp2/3 complex or SH3-domain ligands had no effect. The GEF Vav1, a ligand of HS1 phosphotyrosine, influenced NK cell transendothelial migration. HS1 and Vav1 also affected the speed of NK cells migrating across the surface of the endothelium. We conclude that HS1 has a role in transendothelial migration of NK cells and that HS1 tyrosine phosphorylation may signal through Vav1.

## Introduction

Leukocytes leave the vasculature as part of inflammatory and immune responses. They are recruited to a site of inflammation through a series of steps including capture, rolling, activation, and adhesion, which culminates in migration through the endothelium, termed transendothelial migration (TEM) [1]. The route for TEM may be between endothelial cells (paracellular) or directly through one endothelial cell (transcellular) [2,3]. The paracellular route involves controlled loosening of endothelial cell-cell junctions, creating a space for the leukocyte to travel. The transcellular route requires exquisite control of membrane trafficking, because the endothelial cell creates a channel for the leukocyte while preserving the integrity of its plasma membrane. In both cases, the leukocyte generally squeezes itself through a relatively small hole and passes quickly from one side of the endothelium to the other.

Molecular and cellular analysis of TEM has revealed critical roles for cell adhesion molecules, membrane trafficking and recycling components, and the actin cytoskeleton, under the control of several signaling cascades [4]. Among endothelial cell molecules, ICAM-1 (intercellular adhesion molecule-1) and VCAM-1 (vascular cell adhesion molecule-1) are involved in adhesion of the leukocyte to the endothelial surface through interaction with leukocyte integrins, leading to formation of a “docking structure” for the leukocyte [5]. Other membrane-associated molecules, including PECAM-1 (CD31), CD99, ICAM-2, and JAM family members, play important functional roles in leukocyte transmigration [4]. Leukocytes also have important roles during transendothelial migration [4]. Activation of integrin is required for leukocytes to adhere firmly and extend processes over the surface of the endothelium. β2 and β1 integrins (e.g. CD11a/CD18, CD11b/CD18 and VLA-4) are the primary ones involved [6].

Natural killer cells (NK cells) are large granular lymphocytes and critical components of innate immunity [7,8], providing resistance to infection and cancer. They response rapidly to immune signals, recognizing target cells in the absence of antibodies and MHC class 1 protein. NK cells are distinct from T and B lymphocytes in surface phenotype, target recognition, and function. They lack TCR complex (CD3) expression, and they express N-CAM (CD56) and FcδRIII (CD16) in humans [9]. NK cells also participate in the adaptive immune response, by secreting cytokines and chemokines and by processing antigens [10]. Migration of NK cells to a site of inflammation is initiated by environmental signals, which lead to adhesive interactions between NK cells and vascular endothelial cells, resulting in attachment and transmigration across the endothelium layer [9,11]. NK cells express a number of cell-surface molecules responsible for responding to chemotactic stimuli, binding to the endothelium and TEM [12,13].

NK cell actions during transmigration involve receptor-mediated signaling and cytoskeleton-based processes. HS1 (hematopoietic lineage cell-specific protein 1) can link signaling cascades and actin assembly with generation of force and movement [14]. HS1 is the hematopoietic homolog of cortactin, a prominent Src substrate, which regulates actin dynamics during leading edge formation, invadopodia formation and cell invasion [15]. Cortactin and HS1 bind to Arp2/3 complex and to actin filaments to stabilize branching networks of actin filaments. HS1 contributes to formation of lamellipodia and podosomes, and it regulates T-cell signaling [16–18].

Tyrosine phosphorylation of HS1 plays critical roles in lymphocyte trafficking, antigen-receptor induced T-cell and B-cell signaling and apoptosis [19,20]. Phosphorylation of HS1 can regulate its interaction with other proteins such as the GEF Vav1 [14]. HS1 is a substrate of Syk and Src family kinases. Syk phosphorylation of HS1 on Tyr378 and Tyr397 generates high-affinity binding sites for SH2-domain-containing proteins, which can lead to HS1 tyrosine phosphorylation at position 222 by Src family kinases [21]. In neutrophils, HS1 phosphorylation events on Tyr222, Tyr378, and Tyr397 are required for efficient chemotaxis and for binding of HS1 to Arp2/3 complex [22]. In NK cells, HS1 depletion impairs the formation of lytic synapses and cytolytic activity [14]. HS1 tyrosine phosphorylation at position 397 is important for adhesion of integrins to ICAM-1, lytic synapse formation and cytolytic activity, while phosphorylation of HS1 Tyr378 is important for chemotaxis [14].

HS1 contains an N-terminal Arp2/3-binding region, a repeat region that binds F-actin, a proline-rich region and a C-terminal SH3 domain. Cortactin can synergize with WASp family members to activate Arp2/3 complex [23–25], based on the fact that Arp2/3 complex has two binding sites for acidic/DDW regions [26,27], such as the one found at the N-terminus of cortactin and HS1. The proline-rich region and SH3 domain are important for binding to Vav1, a member of the Dbl family of guanine nucleotide exchange factors (GEF), and to the WIP / WASp heterodimer, which associate with numerous signaling molecules involved in actin cytoskeleton regulation [20].

Here we investigated whether and how HS1 contributes to the process of transendothelial migration by the NK cell. We tested endothelial and NK cell preparations in the presence and absence of a chemoattractant. We used live-cell movies to watch the migration of NK cells over the surface of endothelial monolayers and to follow their movement through the monolayer. Computer-assisted tracking of cell movements and non-parametric statistics of large non-Gaussian data sets facilitated the analysis. We also used conventional transwell assays and staining of fixed preparations. We investigated the importance of HS1’s phosphorylated tyrosine residues, as well as the role of the SH2-containing GEF Vav1, which interacts with phospho-HS1.

## Materials and Methods

Chemicals and reagents were obtained from Fisher Scientific (Pittsburgh, PA) or Sigma-Aldrich (Saint Louis, MO), unless stated otherwise.

### Cell Culture

Cells were cultured at 37°C in 5% CO_2_. Human NK-92 cells (ATCC CRL2407) were obtained from ATCC (Manassas, VA) and cultured in α-Minimum Essential Medium (MEM) base medium supplemented with 12.5% fetal bovine serum (FBS), 12.5% horse serum, myo-inositol, folic acid, β-mercaptoethanol (β-ME), interleukin 2 (IL-2) and penicillin/ streptomycin, according to ATCC recommendations for complete growth medium. Human dermal microvascular endothelial cells (HDMVECs) were obtained from ATCC and cultured in Endothelial Growth Basal Medium (EBM)-2 base medium supplemented with the Microvascular Endothelial Cell Growth Medium-2 (EGM-2 MV) kit with FBS from Lonza (Allendale, NJ).

### Soft Substrate Preparation

20-mm glass-bottom culture dishes (MatTek, Ashland, MA) were coated with aminosilane and activated by treatment with 0.5% glutaraldehyde for 30 min. Polyacrylamide with 0.4% bis-acrylamide was prepared as described [28]. 20 μL of the acrylamide mixture was placed onto an activated cover slip in a glass-bottom dish, and an 18-mm coverslip was placed on top. The acrylamide was allowed to polymerize for 30 min at RT, the top coverslip was removed, and the gel was washed with PBS.

### Cloning and Mutagenesis

HS1 cDNA was obtained from Origene (NM _005335.3) and cloned into the Td-tomato N1 expression vector (gift of Dr. Paul Bridgman, Washington University) at EcoR1 and AgeI restriction sites. Point mutations were introduced into the HS1 expression plasmid by site-directed mutagenesis (QuikChange Kit, Agilent, Santa Clara, CA). First, wild-type (WT) and siRNA-resistant (2,3-Res) HS1 expression plasmids were constructed. Next, individual tyrosine residues were mutated to phenylalanine. Other constructs included a triple point mutant affecting the Arp2/3-binding site, with amino acid residues 18 to 21 changed from DDW to AAA, and an SH3-domain mutant, with amino acid residue 466 Trp changed to Lys. Plasmids are listed in Table 1.

**Table 1:** List of plasmids in this study.

pBJ #	Description of plasmid
2089	tdTom-HS1-WT expression, resistant to shRNA HS1–05
2121	Control shRNA expression, pLKO (Addgene #10879)
2122	HS1–05 shRNA expression, pLKO
2194	tdTom-HS1-WT expression
2339	tdTom-HS1-Y222F expression
2340	tdTom-HS1-Y378F expression
2341	tdTom-HS1-Y397F expression
2342	tdTom-HS1-Y378F/ Y397F expression
2347	tdTom-HS1-Y222F expression, resistant to siRNAs 2 and 3
2348	tdTom-HS1-Y378F expression, resistant to siRNAs 2 and 3
2349	tdTom-HS1-Y397F expression, resistant to siRNAs 2 and 3
2350	tdTom-HS1-Y378F/Y397FsiRNA expression, resistant to siRNAs 2 and 3
2351	tdTom-HS1-DDW (D19A/D20A/W21A) expression
2352	tdTom-HS1-DDW(D19A/D20A/W21A) expression, resistant to siRNAs 2 and 3
2353	tdTom-HS1-W466K expression
2354	tdTom-HS1-W466K expression, resistant to siRNAs 2 and 3
2355	tdTom-HS1-WT expression, resistant to siRNAs 2 and 3

### Antibodies and Immunofluorescence

MatTek dishes were coated overnight at 4°C with ICAM-1 (12.5 μg/mL with 100 μL per 10-mm well). NK cells were treated with 10 μM PMA (phorbol 12-myristate 13-acetate) for 30 min, added to ICAM-1-coated MatTek dishes (1 x 10^5^ cells per well) and incubated for 1 hr at 37°C to allow cells to attach to the glass surface. Cells were fixed and permeabilized in one step, using 4% PFA (paraformaldehye) with 0.3% Triton X-100 in PBS for 10 min. After being washed with PBS, cells were blocked with 5% BSA in PBS and then incubated with primary and secondary antibodies diluted in 3% BSA in PBS. ProLong Gold (Invitrogen, Carlsbad, CA) was used as the mounting agent.

Primary antibodies included rabbit anti-human HS1 (D83A8), rabbit anti-human phospho-Tyr397 HS1, rabbit anti-human VE-cadherin (all from Cell Signaling Technology, Danvers, MA) and mouse anti-human ICAM-1/CD54 (monoclonal antibody BBIG-I1, R&D Systems). Secondary antibodies were Alexa-fluor conjugates (Invitrogen). Alexa-fluor conjugated phalloidin was used for F-actin.

### Assay for Route of Transendothelial Migration

MatTek dishes, with or without polyacrylamide gel, were coated with 10 μg/mL fibronectin in PBS and incubated overnight at 4°C. HDMVEC cells were added and allowed to form a monolayer. After overnight treatment with TNFα (20 ng/mL), the HDMVEC monolayer was washed with HDMVEC media containing 30 ng/mL SDF-1α (stromal cell-derived factor alpha), pre-warmed to 37°C. After several minutes, the SDF-1α-containing medium was removed, and NK cells (1 x 10^5^ NK cells in 1 mL NK cell media) were added onto the HDMVEC monolayer. After incubation for 2 hrs at 37°C, the preparation was fixed and immunostained as described above. This assay was also performed without the chemotactic factor SDF-1α. In this case, NK cells were incubated with the HDMVEC monolayer for 25 min at 37°C.

### HS1 RNAi Targeting and Rescue Expression

A set of four siRNAs (Dharmacon, ON-TARGETplus set of 4) was used to knock down HS1 in NK cells by nucleofection, using the Amaxa cell line nucleofector kit R. Scrambled siRNA was used as a negative control. Each sample included 30 pmol siRNA and 2–3 x 10^6^ cells. Levels of HS1 knockdown in cell lysates were tested by anti-HS1 immunoblot after 72 hrs, with GAPDH as a loading control. HS1 expression plasmids were transfected into NK cells by nucleofection using the same nucleofector kit (up to 2 μg of DNA per sample). To express exogenous HS1 protein while knocking down endogenous HS1, we cotransfected an siRNA-resistant HS1 expression plasmid with the HS1-targeting siRNA. For siRNA 2, codon-neutral mutations were introduced by site-directed mutagenesis, as indicated in lowercase: GAGTaGAaaGAGAcCGAAT. The target sequence of siRNA3, CGGGAAAGTACGTCTAGAT, was in the 3’UTR, so no expression-plasmid mutations were necessary.

To confirm the results with siRNA, we targeted HS1 with shRNA expressed from a pLKO.1 plasmid (pBJ 2122). The targeting sequence, termed HS1-05, was GACACAGATCCTGACTTTG. A control non-hairpin shRNA sequence in the same expression vector served as a negative control (pBJ 2121). To exclude off-target effects, the targeting construct was rescued by expression of a tdTomato-HS1 fusion made resistant to HS1-05 by the following codon-neutral point mutations (lowercase): GAtACtGATCCaGACTTcG (pBJ 2089). The expression plasmid was transfected into NK cells by nucleofection using Amaxa cell line nucleofector kit R. For rescue experiments, the shRNA-resistant tdTomato-HS1 expression plasmid was co-transfected with the shRNA expression plasmid.

For Vav1, a set of four siRNAs (Dharmacon SMARTpooI ON-TARGETplus VAV1 siRNA) was used, as described previously [29,30]. Immunoblots documented decreased levels of Vav1 protein.

### Levels of Expression of HS1 Mutants

To test for levels of expression of HS1 mutants in HS1-knockdown cells, 15 x 10^6^ cells (five cuvettes of 3 x 10^6^ each) were transfected using Amaxa nucleofector kit R. Each cuvette had a total volume of 100 μL, 25 pmol each of HS1 siRNA oligonucleotides 2 and 3, and 2 μg of siRNA-resistant tdTomato-HS1, wild-type or mutant. The amounts were optimized for transfection efficiency in pilot experiments. After 72 hrs, tdTomato-positive cells were selected by flow cytometry sorting. Cells were lysed in 1% NP-40 lysis buffer with the protease inhibitors PMSF, aprotinin, and leupeptin and the phosphatase inhibitor sodium ortho-vanadate. Cell lysates were analyzed by SDS-PAGE and immunoblots.

### Transwell Migration Assay

To assay chemotaxis and transendothelial migration by NK cells, we used transwell chambers (Corning) with 8-μm pore-size inserts. The upper chamber was coated with 10 μg/mL fibronectin in PBS overnight at 4°C and washed with PBS. HDMVEC cells were plated on the filter by adding 0.2 to 0.3 x 10^5^ cells in 100 μL HDMVEC medium. Medium (600 μL) was added to the bottom chamber. After 24 hrs, the medium was replaced with HDMVEC medium containing TNFα (20 ng/mL, 100 μL in the top chamber and 600 μL in the bottom chamber). After incubation for 18 hrs, NK cells (2 x 10^5^ cells in 100 μL NK cell medium) were added to the endothelial monolayer.

NK cells were pretreated by transfection with siRNA targeting HS1 or Vav1 or with siRNA targeting HS1 plus the siRNA-resistant HS1 expression plasmid. For chemotactic migration assays, the medium in the bottom chamber was replaced with medium containing SDF-1α (30 ng/mL). The chambers were incubated for 4 hrs to allow for transmigration. NK cells were collected from the bottom chamber, suspended in 100 μL PBS and counted using a hemocytometer. To test for adequacy of monolayer formation in each transwell apparatus, the filter membrane was excised and fixed and stained with fluorescent phalloidin and anti-VE-cadherin. Results were included only from those transwells where a complete monolayer was observed, without gaps.

### Live-Cell Imaging

We performed live-cell imaging of NK cell migration in preparations with and without treatment by the chemoattractant SDF-1α. For experiments with chemoattractant, HDMVEC monolayers were formed on glass-bottom culture dishes (14 mm, Cat. # P35G-1.5-14-C, MatTek, Ashland, MA) previously coated with fibronectin (10 μg/mL) in PBS overnight at 4°C. HDMVECs (1 x 10^5^) in HDMVEC medium were added to each dish and placed in culture overnight. The monolayers were treated with TNFα (20 ng/mL) overnight. During the same time period, NK cells were transfected with siRNAs targeting HS1 and / or Vav1 for 72 hrs.

The endothelial monolayer was washed with HDMVEC medium containing 30 ng/mL SDF-1α, pre-warmed to 37°C. After several minutes, the SDF-1α-containing medium was removed, and siRNA-treated NK cells were added to the monolayer and incubated for 1 hr. Preparations were imaged using a Zeiss LSM 510 confocal microscope with a 40X 1.2 NA objective and an environmental chamber. DIC images were captured for 1 hr at 1-min intervals as a stack of three z-axis focal planes (1 μm thick), which included the endothelial surface and the NK cells. Maximum intensity projections of the z-axis focal planes were used to observe and measure the rate of NK cell migration on the endothelial monolayer.

To observe the migration of NK cells in the absence of SDF-1α stimulation, the methods were similar, with minor differences. Glass-bottom culture dishes were coated with 15 μg/mL fibronectin overnight and washed with cold PBS. To capture live cell images, HDMVEC medium was replaced with NK cell medium, the culture dish was placed on the incubator stage, and an area of the endothelial monolayer without gaps was identified. NK cells (1 x 10^5^) were added, and images were captured immediately after the cells settled down onto the endothelial monolayer. Images were collected every 20 s for 1 hr, with a 10X objective. We used a spinning-disc confocal microscope (IX73, Olympus Corporation, Japan) with a stage device to maintain cells at 37°C and 5% CO_2_ (Tokai Hit CO., Ltd, Japan).

### Quantitative Analysis of Cell Movements in Movies

To quantify the migration results and analyze the data, computer-assisted tracking of cell position was performed. We collected the x-y coordinates of the front of NK cells over time, using MTrackJ, developed by Meijering and colleagues [31] (available online at http://www.imagescience.org/meijering/software/mtrackj.) We tracked all the cells in a given movie, without any selection. Migration rates were calculated as the change in distance per unit time (μm/min). We calculated instantaneous speed (distance per time for each frame of every tracked cell), total path length per time for each cell track, and net displacement (distance from start to finish) per time for each cell track. Persistence, defined as net displacement divided by path length, was calculated for the entirety of one cell track and also in smaller windows of time that were moved over each track from beginning to end.

Data were analyzed and statistics performed using GraphPad Prism. Distributions of speeds were far from Gaussian, so data were analyzed as medians and 95% confidence intervals. Statistical tests of significance were unpaired and two-tailed, generating p values from Kolmogorov-Smirnov and Mann-Whitney tests as implemented in Prism.

An observer counted TEM events in the movie, scoring NK cells that moved underneath the monolayer. The total number of NK cells on the monolayer was counted, and percentage of TEM was calculated as the number of TEM events divided by the number of NK cells. The accumulated number of TEM events versus time was also plotted, based on the movie frame when a cell began to transmigrate. Cells that transmigrated back up to the top surface of the endothelial monolayer were not counted.

### Protein Pull-down Assays

Total proteins were extracted from control and HS1-siRNA-treated NK cells by lysing cells in IP buffer (20 mM Tris, 10 mM EDTA, 1 mM EGTA, 150 mM NaCl, 0.05% Triton X-100 and 0.05% NP40) containing PMSF and protease inhibitor cocktail (complete, Mini, EDTA free tablet, Roche). Lysates were centrifuged at 15,000 x g for 10 min at 4°C, and supernatants were collected. To test whether HS1 knockdown inhibited Vav1 pull-down, we incubated protein A beads coated with anti-HS1 with control or HS1-depleted NK cell lysates overnight at 4°C. After washing, the beads were boiled with SDS-PAGE sample buffer. Samples were electrophoresed on 10% SDS polyacrylamide gels, followed by immunoblotting with antibodies to HS1 (rabbit anti-human HS1, Cell Signaling) or Vav1 (rabbit anti-Vav1, Cell Signaling).

## Results

### HS1 and TEM of NK Cells in Transwell Assays

**Fig 1:**
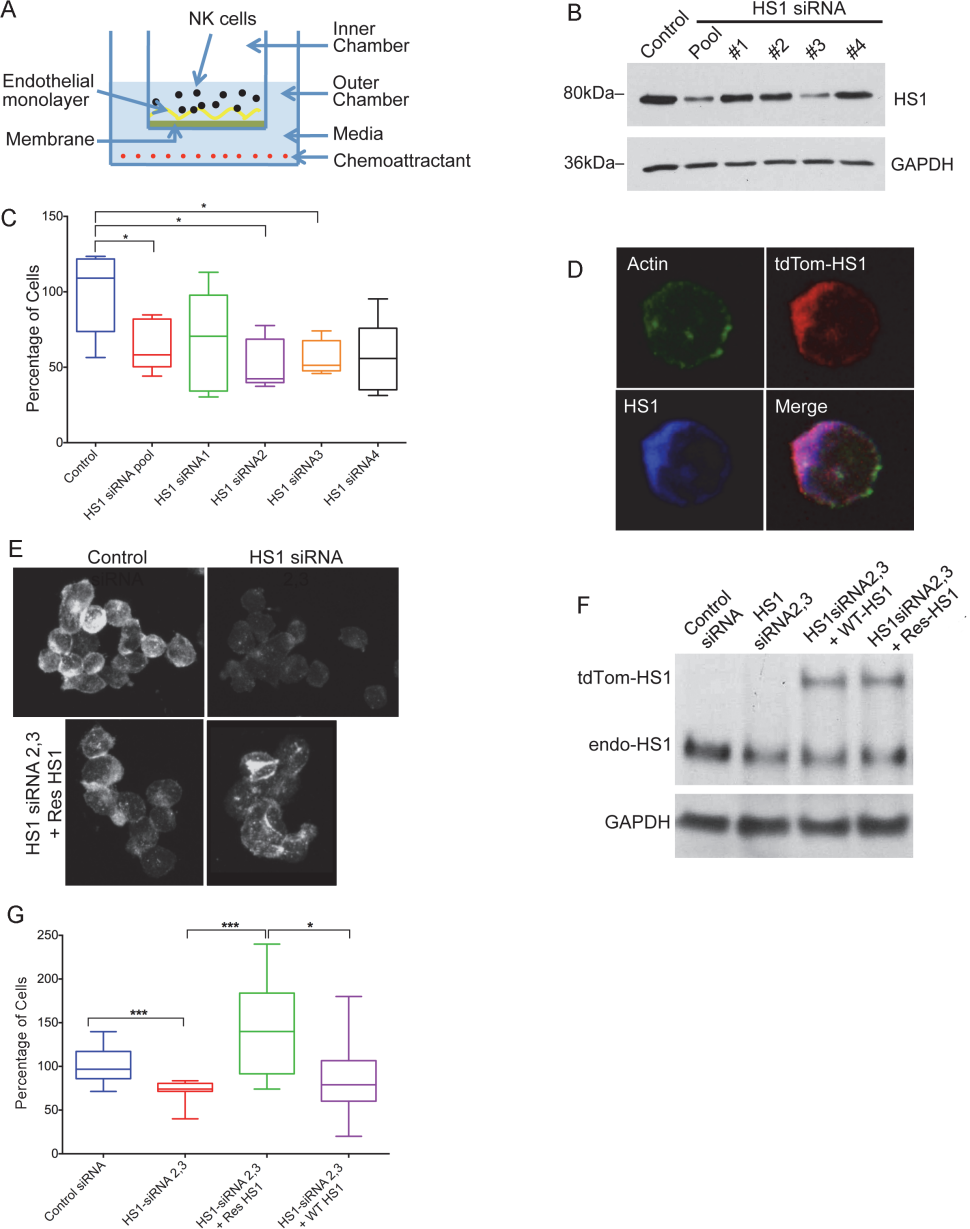
HS1 and TEM of NK cells in transwell assays. A) Diagram of transendothelial migration assay in a transwell device. B) Depletion of HS1 protein by siRNA, shown by immunoblot after 72 hrs. NK cells were treated with a pool of four siRNAs or one of the four. GAPDH is a loading control. C) Effects of HS1 knockdown on TEM. Plotted values are number of cells in the lower chamber, as a percentage of the mean of the control sample value on each day. Box-and-whisker plots (box: 25th to 75th percentiles, whiskers: min to max, middle line: median). Asterisks indicate statistical significance (*P<0.05. Unpaired Student’s t-test, n = 5 for each condition.) D) Fluorescence micrographs of NK cells, showing expression and co-localization of expressed HS1-tdTomato (red), F-actin (green, Alexa Fluor 488 phalloidin), and total HS1, including endogenous (blue, anti-HS1 staining). E) Fluorescence micrographs of NK cells stained with anti-HS1 to show siRNA-induced depletion of HS1 and expression of siRNA-resistant HS1 protein. F) Expression of siRNA-resistant HS1 in NK cells knocked down for HS1 with siRNA, shown by immunoblot with anti-HS1. Knockdown used a combination of HS1 siRNAs 2 and 3. G) Rescue of TEM phenotype in HS1-knockdown NK cells by expression of HS1. Cells as in panels E and F. Number of cells in the lower chamber, as a percentage of the mean of the control sample value on each day, with box-and-whisker plots as in panel C. Asterisks indicate statistical significance. (* P< 0.05, *** P < 0.0005. Unpaired Student’s t-test, N = 9–12 experiments for each condition.)

To investigate the role of HS1 in TEM by NK cells, we depleted HS1 from NK cells using RNAi. As an initial approach, we assayed TEM using traditional transwell culture chambers with porous filters, as illustrated in Fig. 1A. NK cells added to an endothelial monolayer were challenged to migrate across the monolayer towards a chemoattractant, SDF-1α. Cells that crossed the monolayer were collected and counted at points over time.

To deplete HS1 from NK cells, we tested four siRNA oligonucleotides, singly and as a pool. siRNA 2, siRNA 3 and the pool of four caused substantial depletion of HS1 (69%), assayed by immunoblot (Fig. 1B). siRNA 4 produced a small effect, and siRNA 1 had no effect. In the transwell assay, HS1 depletion decreased the number of cells in the lower chamber, compared with control (Fig. 1C). siRNA 2, siRNA 3 and the pool caused statistically significant decreases, but siRNA 1 and siRNA 4 did not, consistent with the decreases in the level of HS1 protein. For subsequent experiments, we combined siRNA 2 and siRNA 3, and the extent of HS1 depletion was 70–75% by immunoblot.

To document specificity for HS1 and to test HS1 mutants, we constructed tdTomato-HS1 expression plasmids resistant to siRNA 2 and siRNA 3. First, we tested tdTomato-HS1 for expression and localization. The fusion protein was expressed, and it localized to F-actin (Fig. 1D). Next, we tested expression of tdTomato-HS1 for rescue of the transwell knockdown phenotype. Levels of HS1 in knockdown and expression-rescue cells, assessed by anti-HS1 immunofluorescence (Fig. 1E) and immunoblot (Fig. 1F, S1 Fig.), were comparable to those in control cells.

In the TEM transwell assay (Fig. 1G), siRNA-resistant HS1 (Res HS1, green) fully rescued the defect caused by HS1 knockdown (red), with levels exceeding those of control cells (blue), by a statistically significant margin (p = 0.02) (Fig. 1G). The non-siRNA-resistant construct (WT HS1, purple) produced a smaller increase in the level, which was not statistically significant compared to HS1 knockdown (p = 0.28). The levels of rescue for the two wild-type constructs, siRNA-resistant and not siRNA-resistant, were consistent with the level of HS1 protein. We conclude that the level of HS1 is a key parameter controlling the ability of NK cells to perform TEM in the transwell assay in preparations treated with SDF-1α.

To confirm these results with an independent RNAi approach, we targeted HS1 with shRNA expressed from a plasmid (S2 Fig.). Overall, similar results were obtained. HS1-depleted NK cells showed decreased migration into the lower chamber in the transwell assay, compared to control, and the phenotype was rescued by expression of HS1 resistant to the HS1-targeting shRNA.

### HS1 and Migration of NK Cells across the Surface of the Endothelium

**Fig 2:**
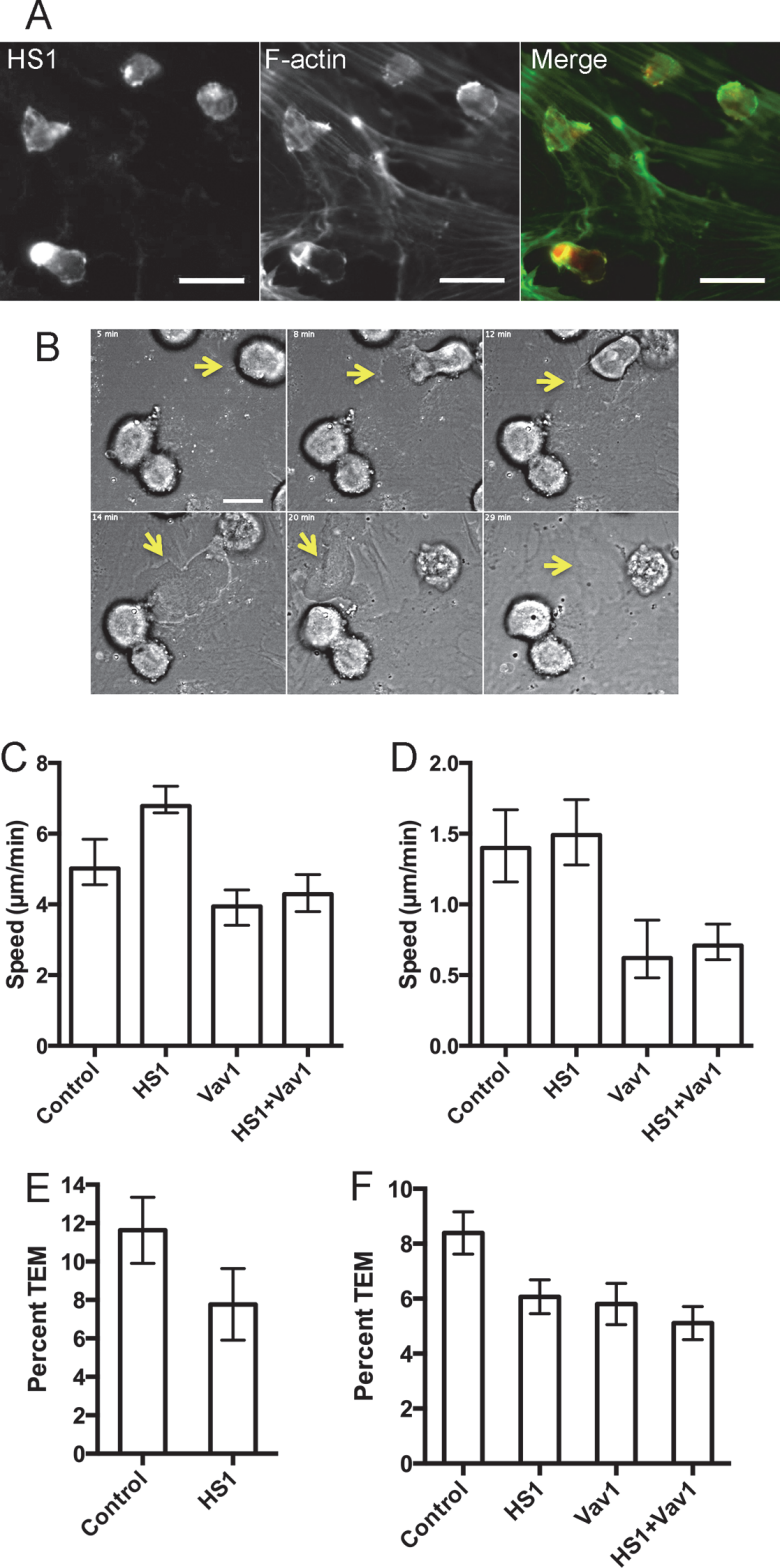
TEM events by NK cells on HDMVEC monolayers based on live-cell movie analysis. A) Endogenous HS1 (red) and F-actin (green) in NK cells migrating on the surface of HDMVEC monolayer observed by anti-HS1 and phalloidin fluorescence. Scale bar = 20 μm. B) DIC images from a movie (S2 Movie), illustrating how the passage of the NK cell through the endothelial monolayer leaves a defect. Scale bar = 10 μm. C) Speed of cell migration, based on path length. Median and 95% confidence intervals are plotted. Data from Table 4-1. D) Speed of cell migration, based on net displacement. Median and 95% confidence intervals are plotted. Data from Table 4-2. E) Percentage of TEM events from movie analysis with SDF-1α. The number of TEM events as a percentage of the total number of NK cells on the surface of the endothelial monolayer in the first frame. Error bars are standard error of proportion. The difference is not statistically significant by z-test (p = 0.15) or by Fisher’s exact test for a 2 x 2 contingency table (p = 0.19). Data combined from two or three experiments per day on three days. F) Percentage of TEM events from movie analysis without SDF-1α. The number of TEM events as a percentage of the total number of NK cells on the surface of the endothelial monolayer in the first frame. Error bars are standard error of proportion. The differences between control and depleted-cell values are statistically significant with p values of 0.022, 0.024 and 0.001 for HS-depleted, Vav1-depleted and HS1+Vav1-depleted NK cells, based on chi-square tests with Yates’ correction. No other differences are statistically significant. Data combined from experiments on three days.

To investigate the role of HS1 in the individual steps that compose the process of TEM, we turned from transwell assays to direct visualization by light microscopy. When NK cells encounter the surface of the endothelium, they adhere and migrate across the surface, as illustrated in movies in S1 Movie and S2 Movie. Dynamic actin filaments and multiple actin regulatory proteins are enriched at the leading edge of migrating cells. Localization of HS1 and F-actin in NK cells migrating on the surface of endothelial monolayers revealed slight enrichment at the leading edge, based on fluorescence staining (Fig. 2A).

To test the functional importance of HS1 for NK-cell migration, we depleted HS1 with siRNAs and collected movies of NK cells migrating across the endothelial surface (Examples in Fig. 2B, S1 Movie and S2 Movie). We used two slightly different experimental protocols. In one set of experiments, the endothelial monolayer was incubated with SDF-1α for several minutes, then NK cells were added and allowed to interact with the monolayer for 1 hr before movies were collected. In a second set of experiments, SDF-1αwas not included, and movies were collected immediately, as soon as the NK cells settled onto the endothelial monolayer.

We tracked the positions of the NK cells over time, and we analyzed the movement in several ways. To avoid selection bias, a computer-based tracking program followed all the cells over time. We calculated the frame-to-frame distance moved by each cell in each track, the distance from start to finish of each cell track (net displacement), and the total distance moved by each cell along its track (path length). From these distances, we calculated speeds.

For “instantaneous” speeds, calculated from frame-to-frame distance, the time interval was sufficiently small that many distances were at or near zero, with a non-Gaussian distribution extending to higher values. Histograms of the value reveal that HS1 depletion had no significant effect, in either set of experiments. S3 Fig. shows representative results, from the protocol without SDF-1α treatment.

For speeds based on path length, the two sets of experiments revealed different effects of HS1 depletion, which were modest but achieved statistical significance owing to the large amount of data (Table 2-1, 2-2). The distribution of speeds was non-Gaussian, so the results are listed in the tables as medians with 95% confidence intervals, and p values were calculated with two non-parametric tests – Kolmogorov-Smirnov (KS) and Mann-Whitney (MW). With SDF1-α treatment, HS1 depletion produced a modest (20%) decrease in speed, with non-overlapping 95% confidence intervals and p values < 0.01 (Table 2-1). However, with untreated cells, HS1 depletion led to an increase in speed, by a slightly larger amount (34%), with non-overlapping 95% confidence intervals and p values < 0.0001 (Table 2-2, Fig. 2C). In each case, the number of data points (N in the tables) was large, which accounted for the differences achieving statistical significance. The differences were reproducible and consistent, observed on each of three different days in each set of experiments.

**Table 2:** Migration Speeds for NK Cells.

Table 2-1. Preparations treated with SDF-1α.			
	Median	95% CI	N
Control	2.94	2.62–3.11	314
HS1-depleted	2.34	2.22–2.55	272
Non-parametric p values			
Kolmogorov-Smirnov test		0.0051	
Mann-Whitney test		0.0065	

Table 2-2. Preparations not treated with SDF-1α.			
	Median	95% CI	N
Control	5.02	4.56–5.84	393
HS1-depleted	6.78	6.59–7.34	436
Non-parametric p values			
Kolmogorov-Smirnov test		< 0.0001	
Mann-Whitney test		< 0.0001	

Speed (μm/min) defined as total displacement (path length) divided by time for control vs HS1-depleted cells. The distributions are not Gaussian, so the values listed are median, 95% confidence interval of the median and number of data points (N). P values from two non-parametric tests of significance are listed. Data include tracks for all cells in separate experiments on three different days.

For speeds based on net displacement (start to finish), modest differences in the same direction were observed, but they failed to achieve statistical significance for the most part (Table 3-1 and III-2). Again, the distributions were not Gaussian, and non-parametric statistical tests were performed. With SDF1-α treatment, HS1 depletion produced a modest (22%) decrease in speed, but the 95% confidence intervals overlapped, and the p values were 0.03 and 0.08 from KS and MW tests, respectively (Table 3-1). Results from three days were similar. With untreated cells, HS1 depletion again led an increase in speed, as found for path-length speeds, but only by a small amount (6%), with overlapping 95% confidence intervals and p values of 0.8 and 0.3 (Table 3-2, Fig. 2D). Moreover, the results on different days were not consistent; two days showed small decreases and one day showed a larger increase. Overall, this analysis revealed no significant difference between control and HS1-depleted cells; however, the trends were in the same direction as those for the path-length speeds.

**Table 3:** Migration Speeds for NK Cells.

Table 3-1. Preparations treated with SDF-1α.			
	Median	95% CI	N
Control	0.96	0.83–1.06	282
HS1-depleted	0.75	0.61–0.93	272
Non-parametric p values			
Kolmogorov-Smirnov test		0.033	
Mann-Whitney test		0.078	

Table 3-2. Preparations not treated with SDF-1α.			
	Median	95% CI	N
Control	1.40	1.16–1.67	393
HS1-depleted	1.49	1.28–1.74	436
Non-parametric p values			
Kolmogorov-Smirnov test		0.81	
Mann-Whitney test		0.31	

Speed (μm/min) defined as net displacement (distance start to finish) divided by time for control vs HS1-depleted cells. The distributions are not Gaussian, so the values listed are median, 95% confidence interval of the median and number of data points (N). P values from two non-parametric tests of significance are listed. Data include tracks for all cells in separate experiments on three different days.

Finally, we calculated persistence, defined as net displacement divided by path length. Persistence values vary from zero to one, and persistence can be calculated for part or all of a track. We considered the possibility that one cell track might include segments with large movement and high persistence along with segments with little to no movement and low persistence. Therefore, we calculated persistence for sliding windows of 5, 10, 15, and 30 frames, as well as the entire track, for each cell track. Values from zero to one were found, and smaller windows gave higher values, as expected. Only small differences between HS1-depleted and control cells were observed. The differences were not consistent within one day or between days. Results from a representative experiment are shown in S4 Fig. Overall, persistence analyses revealed no difference between control and HS1-depleted cells.

### HS1 Depletion Affects the Route and Overall Efficiency of TEM

Next, we asked whether HS1 had a role in the ability of NK cells to perform TEM. First, we observed TEM directly in the live-cell movies used to analyze migration above. NK cells on the upper surface of the endothelium stopped, squeezed through the endothelium, and appeared below the endothelium (Fig. 2B, S2 Movie). We counted these events and calculated their percentage relative to the total number of NK cells.

For SDF-1α-treated preparations, the frequency of TEM was 11.6% (40 TEM events / 344 cells) for control cells and 7.8% (16/206) for HS1-depleted cells (Fig. 2E). The difference was not statistically significant. For non-treated preparations, the frequency of TEM was 8.4% (108/1297) for control cells and 6.1% (90/1482) for HS1-depleted cells (Fig. 2F). The differences between the control and the three depleted-cell values were statistically significant, but the differences among the depleted-cell values were not.

**Fig 3:**
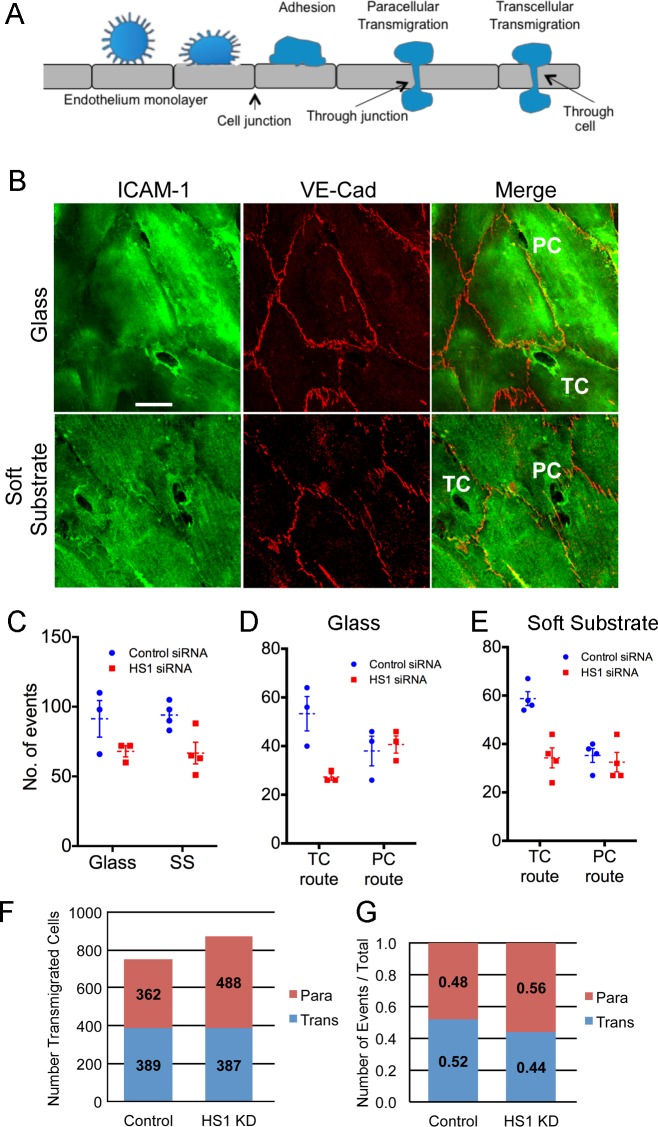
Transcellular vs paracellular route of TEM. A) Diagram illustrating paracellular and transcellular routes. B) Representative confocal fluorescence images of cells taking the paracellular (PC) and transcellular (TC) routes. The migrating NK cells appear as small dark holes surrounded by intense anti-ICAM-1 staining, and the endothelial cell-cell junctions are visualized by anti-VE-cadherin staining. The endothelial cell substrate was glass in the upper panel and soft substrate (polyacrylamide) in the lower panel. Scale bar = 10 μm. C) Total number of transendothelial migration events, with the endothelial monolayer on glass or soft substrate (SS). D) Numbers of paracellular and transcellular transmigration events on glass. E) Numbers of paracellular and transcellular transmigration events on soft substrate. For panels D to F, each plotted data point represents the average of three values from one experiment. The mean and standard error of the values of the plotted points are also indicated, by the dotted lines and error bars. HDMVEC cells were washed with SDF-1α-containing media before the addition of NK cells, and NK cells were incubated for 2 hrs on the monolayer before fixation. F and G) Transendothelial migration events and routes. NK cells were incubated for 25 min on the surface of an HDMVEC monolayer. NK cells migrated via transcellular and paracellular routes were counted over entire slide. The data are based on experiments in triplicate on two different days. F) Numbers of events. G) Ratios. The difference in the ratio of transcellular to paracellular events between control and HS1 knockdown is small but statistically significant because the number of data points is large. Based on a 2 x 2 contingency table, Fisher’s exact two-tailed p-value is 0.0024.

Using an independent approach, we fixed and stained the preparations, which allows one to observe many fields of view and to determine whether cells migrate via a paracellular route or a transcellular route (Fig. 3A). The route is revealed by the position of the NK cell with respect to endothelial cell junctions, which are visualized with anti-ICAM-1 and anti-VE-cadherin antibody staining, respectively (Fig. 3B).

First, in experiments with SDF-1α treatment, HS1 depletion led to modest decreases in the total number of TEM events, for endothelial monolayers on glass or soft substrate (Fig. 3C). HS1 depletion decreased the frequency of the transcellular route but not the paracellular route for both glass (Fig. 3D) and soft substrate (Fig. 3E). Second, in experiments without SDF-1α, the ratio of transcellular to paracellular route events was also decreased with HS1 depletion; however, the difference was small and only achieved statistical significance because of the large number of data points (Fig. 3G). In these experiments, HS1 depletion led to a small non-significant increase in the number of TEM events (Fig. 3F).

### Tyrosine phosphorylation of HS1

Tyrosine phosphorylation of HS1 is known to be important for many of its cellular functions [16–18]. In NK cells, HS1 Tyr 397 is required for cell adhesion to ICAM-1 and for target-cell killing, and Tyr 378 is required for chemotaxis [14]. In neutrophils, phosphorylation of HS1 Tyr 222 is important for chemotaxis [22]. We investigated the role of the three HS1 tyrosine residues, 222, 378 and 397, in NK cells using transwell assays of TEM.

**Fig 4:**
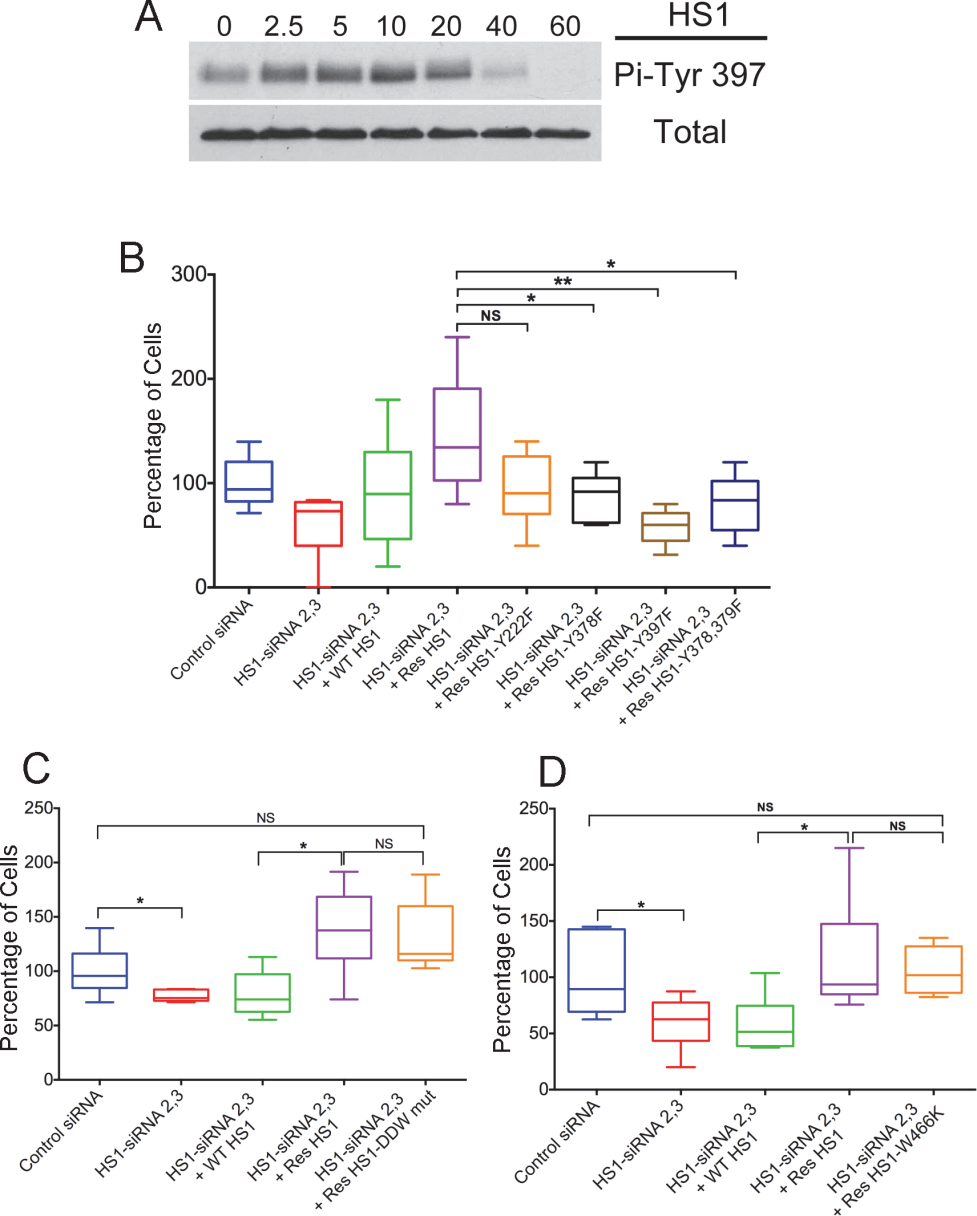
Expression Rescue of HS1 Mutants in HS1-depleted NK Cells. A) Phosphorylation of HS1 Tyr397 in response to SDF-1α. Immunoblots probed with anti-Phospho-Tyr397 and anti-HS1. NK cells (5 x 10^6^) treated with SDF-1α (30 ng/mL) for the indicated times (min). B—D) Function of HS1 mutants in TEM by transwell assay. Number of cells in the lower chamber, as a percentage of the mean of the control sample value on each day, with box and whisker plots. Boxes: 25th to 75th percentiles; whiskers: minimum and maximum values. B) Mutations of phosphorylated tyrosine residues. Compared to control siRNA (blue), HS1 depletion by siRNA causes decreased TEM (red), and the defect is rescued by expression of wild-type HS1 (green) or siRNA-resistant wild-type HS1 (purple). Expression of siRNA-resistant forms of single-mutant HS1 Y378F (black), single-mutant HS1 Y397F (brown) or double-mutant HS1 Y378F Y397F (dark blue) does not rescue the defect, comparing their values to the value for siRNA-resistant wild-type (purple). Expression of siRNA-resistant HS1 Y222F (orange) rescues with a value that is slightly less, but not statistically significant, from that of siRNA-resistant wild type. Asterisks indicate *P>0.05, **P>0.005 (unpaired Student’s t-test, N = 6–9). C) Mutation of Arp2/3 complex binding site. Expression of siRNA-resistant HS1 with mutation of DDW residues to AAA (orange) rescues the defect, with no difference compared to siRNA-resistant wild-type HS1. N = 6 in each case. D) Mutation of SH3 domain at ligand-binding site. Expression of siRNA-resistant HS1 with the mutation W466K (orange) rescues the defect, with no difference compared to siRNA-resistant wild-type HS1. n = 6 in each case.

First, we showed that HS1 was phosphorylated on Tyr in our NK cell preparations treated with SDF-1α (Fig. 4A). Immunoblots with anti-Pi-Tyr397 showed a low basal level of phosphorylation, with increased levels at 10 min, followed by a decrease to below basal levels at 60 min.

Next, HS1 was depleted from NK cells as above, by HS1 siRNA, and siRNA-resistant HS1 mutants were expressed and tested for the ability to rescue the TEM phenotype in transwell assays. Immunoblots confirmed that endogenous HS1 was depleted and that the mutants were expressed at comparable levels (S1 Fig.). Wild-type siRNA-resistant HS1 rescued the phenotype, as discussed above (Fig. 1G, reproduced on the left side of Fig 4B). In contrast, Y378F and Y397F single point mutants of HS1, expressed in siRNA-resistant constructs, failed to rescue (Fig. 4B, purple vs black and brown, respectively). A Y378F / Y397F double point mutant also failed to rescue; the result was similar to those for the single point mutants (Fig. 4B, dark purple), suggesting that both tyrosine residues are necessary for the same pathway. For the Y222F single point mutant, the level of rescue was similar to that of the other point mutants; however, the result was not statistically significant compared to wild-type (Fig. 4B, orange vs purple). Together, the results indicate that HS1 tyrosine residues play important roles in NK cell TEM as measured by transwell assays.

### Role for the Phospho-HS1 ligand Vav1

**Fig 5:**
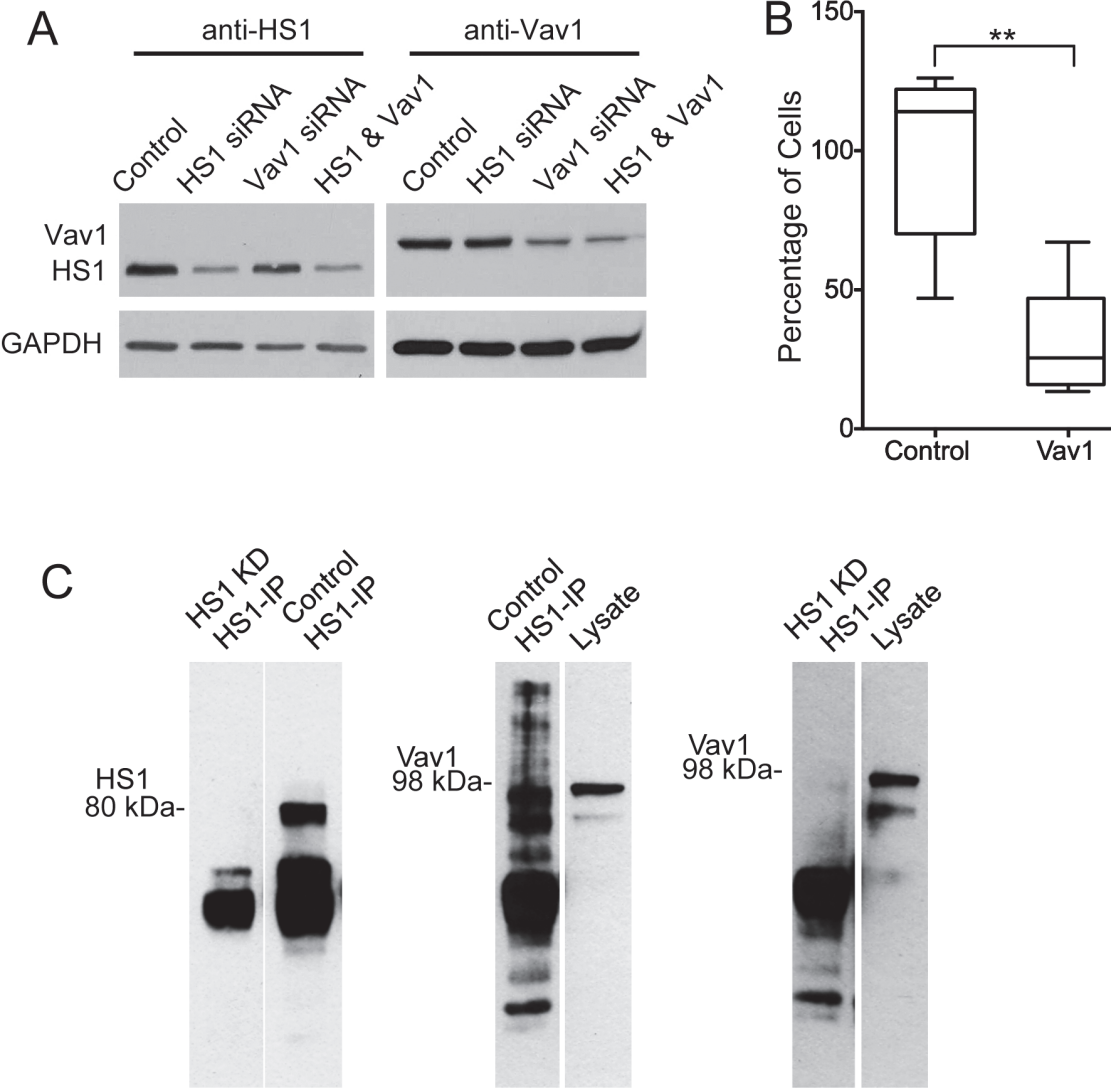
Role of Vav1 in TEM by NK cells. A) Immunoblots with anti-HS1 and anti-Vav1 showing depletion of HS1 and Vav1 after 72 hrs of siRNA treatment. B) Decrease in TEM in transwell assay by NK cells treated with Vav1 siRNA, compared to control siRNA. Number of cells in the lower chamber, as a percentage of the mean of the control sample value on each day, with box and whisker plots. Boxes: 25th to 75th percentiles; whiskers: minimum and maximum values. N = 6. Asterisks indicate **P<0.005 (unpaired Student’s t-test). C) Left panel: Immunoblot with anti-HS1. The left lane shows the absence of HS1 in an anti-HS1 immunoprecipitate from a whole-cell lysate of NK cells treated with siRNA targeting HS1. The right lane shows the result for cells treated with control siRNA. Middle panel: Immunoblot with anti-Vav1. The left lane shows the presence of Vav1 protein in an anti-HS1 precipitate from a lysate of NK cells treated with control siRNA. The right lane shows the presence of Vav1 in the lysate. Right panel: Similar to the middle panel, except with a lysate from NK cells depleted for HS1.

Vav1, a GEF for the Rho family of GTP binding proteins, is a potential ligand for phosphorylated tyrosine residues of HS1, because Vav1 is known to bind HS1 and to require the Tyr 397 residue [17]. To investigate a role for Vav1 in NK cell TEM, we depleted Vav1 by siRNA and performed transwell assays as above. Depletion of Vav1 protein was confirmed by immunoblot (Fig. 5A). The level of transmigration for Vav1-depleted NK cells was far less than the number for control cells, by more than half (Fig. 5B). Thus, Vav1 may act as an effector downstream of HS1 in NK cells, helping to transmit or amplify signals required for TEM.

We assayed the effect of Vav1 depletion on NK cell migration across the surface of the endothelial monolayer, as part of movie-based experiments testing the effect of HS1 depletion described above. Simultaneous depletion of both HS1 and Vav1 was also tested. Speeds were decreased for Vav1-depleted cells, calculated from path length (Fig. 2C, Table 4-1) and net displacement (Fig. 2D, Table 4-2), in contrast to the increases observed for HS1 depletion. Depletion of both HS1 and Vav1 produced intermediate values (Fig. 2C and 2D, Table 4-1 and 4-2).

**Table 4:** Effect of Vav1 and HS1 plus Vav1 Depletion on Migration Speeds for NK Cells.

Table 4-1. Speed (μm/min) defined as total displacement (path length) divided by time. Values for Control and HS1-depleted cells are the same as those in Table 2-2.				
	Median	95% CI	N	
Control	5.02	4.56–5.84	393	
HS1	6.78	6.59–7.34	436	
Vav1	3.94	3.41–4.41	304	
HS1+Vav1	4.29	3.80–4.84	401	
Non-parametric p values				
Kolmogorov-Smirnov test		Control	HS1	Vav1
	HS1	<0.0001		
	Vav1	<0.0001	<0.0001	
	HS1+Vav1	0.0026	<0.0001	0.013
Mann-Whitney test		Control	HS1	Vav1
	HS1	<0.0001		
	Vav1	0.0007	<0.0001	
	HS1+Vav1	0.0582	<0.0001	0.30
Table 4-2. Speed (μm/min) defined as net displacement (distance start to finish) divided by time. Values for Control and HS1-depleted cells are the same as those in Table 3-2.				
	Median	95% CI	N	
Control	1.40	1.16–1.67	393	
HS1	1.49	1.28–1.74	436	
Vav1	0.62	0.48–0.89	304	
HS1+Vav1	0.71	0.61–0.86	401	
Non-parametric p values				
Kolmogorov-Smirnov test		Control	HS1	Vav1
	HS1	0.81		
	Vav1	<0.0001	<0.0001	
	HS1+Vav1	< 0.0001	<0.0001	0.74
Mann-Whitney test		Control	HS1	Vav1
	HS1	0.31		
	Vav1	0.0001	<0.0001	
	HS1+Vav1	<0.0001	<0.0001	0.76

Preparations not treated with SDF-1α. Combination of all tracks from experiments on three days. The distributions are not Gaussian, so the values listed are median, 95% confidence interval of the median and number of data points (N). P values from two non-parametric tests of significance are listed. Data include tracks for all cells in separate experiments on three different days.

Frame-to-frame “instantaneous” speeds showed no effect of depletion of Vav1 or HS1 plus Vav1 (S3 Fig.), as found for depletion of HS1. Persistence values were also not affected.

We examined the effect of Vav1 depletion on TEM by scoring events in live-cell movies (Fig. 2F). Depletion of HS1 and Vav1 had similar effects, decreasing the level of TEM, and the double knockdown had a slightly larger effect. All the values for depletion samples were different from control by statistically significant amounts, but the differences between double and single knockdowns were not significant. Based on this result with this assay, HS1 and Vav1 appear to function in the same pathway.

To confirm that HS1 interacts with Vav1 in the NK cell system, we assayed for co-precipitation of Vav1 with HS1 (Fig. 5C). HS1 was precipitated with anti-HS1, and Vav1 was detected by immunoblot (middle panel, Fig. 5C). In a control experiment, with cells depleted of HS1 by siRNA, Vav1 and HS1 were not observed in the immunoblots (left and right panels, Fig. 5C).

### Arp2/3-Binding and SH3 Domains of HS1

The N-terminal domain of HS1 has an acidic/DDW region, which binds to Arp2/3 complex, and the C-terminus has an SH3 domain [18]. We tested the importance of these domains by testing for rescue of the HS1-knockdown transwell phenotype with expression of HS1 mutants. For Arp2/3 binding, we changed the DDW residues to AAA, and for SH3 binding, we changed Trp 466 to Lys. The DDW to AAA mutant of HS1 cannot bind Arp2/3 complex and changing Trp 466 to Lys in the SH3 domain of cortactin inhibits ligand binding [32]. Both mutants rescued the HS1-depletion phenotype in the transwell assay, similar to wild-type HS1 (Fig. 4C and 4D, respectively). Thus, we see no evidence of a critical role for HS1 binding to Arp2/3 or SH3-domain ligands in NK cells using this assay.

## Discussion

HS1 is known to have an important role in NK cells in the processes of chemotaxis, cell adhesion, actin assembly at the lytic synapse and target cell lysis [14]. The multi-step process of TEM includes a number of similar motility-related phenomena. Therefore, we investigated whether NK-cell HS1 has an important role in TEM.

### Role for HS1 in TEM

First, we found that depletion of HS1 decreased the frequency of transmigration events by NK cells on endothelial monolayers using conventional transwell assays. The knockdown phenotype was specific for HS1, and similar effects were found using two different RNAi approaches. The effect was a moderate one, with HS1-depleted NK cells showing approximately half the level of migration of cells into the lower chamber of the transwell apparatus.

However, the transwell assay involves chemotaxis, because the NK cells are induced to migrate through the transwell filter, from the upper to lower chamber, by a chemoattractant. Therefore, we observed transendothelial migration directly, using microscopy of living and fixed cell preparations. We performed those experiments in two ways—with and without treatment of the cell preparations with a chemoattractant, SDF-1α.

In movies of living cells, TEM events were counted by direct observation. Small to moderate decreases in TEM were observed for HS1 depletion (Fig. 2D, 2E). The decrease in TEM with HS1 depletion was statistically significant without but not with SDF-1α. An alternative approach was antibody staining of fixed preparations. Using this approach, we observed a moderate decrease in TEM events for HS1 depletion with SDF-1α (Fig. 3D) but not without (Fig. 3G).

### Transcellular vs Paracellular Route for TEM

We found that HS1 depletion caused NK cells to prefer the paracellular route relative to the transcellular route for TEM. For preparations treated with SDF-1α, the number of transcellular-route events decreased while the number of paracellular-route events did not change (Fig. 3E, F). For untreated preparations, the number of transcellular-route events did not change but the number of paracellular events increased (Fig. 3G). In both cases, the transcellular to paracellular ratio decreased with HS1 depletion. The molecular mechanisms for the two routes may differ substantially. Cell junction proteins play dominant roles in the paracellular route, and integrin-based adhesion molecules and receptors are critical for the transcellular route [3].

### Migration of NK Cells on the Surface of the Endothelial Monolayer

In movies of living cells, we observed NK cells migrating over the surface of the endothelial monolayer, before they executed TEM. Computer-assisted tracking of many cells provided us with large quantities of data that revealed several moderate statistically significant differences. Depletion of HS1 led to increased rates of NK cell migration, based on measurements of path length and net displacement (Fig. 2B, C). This result is unexpected if one assumes that HS1 promotes actin assembly and actin assembly is necessary for cell migration. However, the biochemical effects of HS1 on actin assembly are complex. HS1 binds directly to F-actin, and it binds and activates Arp2/3 complex; together, these interactions stabilize the branches of the actin filament network. Several other acidic/DDW regulators can bind to Arp2/3 complex, in a 2:1 stoichiometry [23,26,27]. The roles of individual regulators remain to be defined, especially in the complex environment of the cell, as revealed by systematic mutational studies in budding and fission yeast [33–35].

### HS1 Tyrosine Phosphorylation and the GEF Vav1

HS1, like its homolog cortactin, is a prominent substrate for tyrosine phosphorylation downstream of Src. We found that tyrosine phosphorylation of HS1 is also important for NK-cell TEM, based on failure of tyrosine to phenylalanine mutants to rescue the depletion phenotype in the transwell assay. Tyr residues 378 and 397, which have been implicated previously in NK-cell function, were both important [14]. The Tyr 222 mutant also failed to rescue, but quantitative analysis did not show statistical significance, limiting the strength of the conclusion.

Phospho-HS1 interacts with the SH2 domain of the GEF Vav1 [17]. We confirmed that HS1 and Vav1 interact in our NK cell preparations, based on co-precipitation. siRNA-induced depletion of Vav1 decreased the efficiency of TEM in transwell assays (Fig. 5B) and movie-based assays (Fig. 2E). The double depletion of Vav1 and HS1 did not decrease TEM more than either of the single depletions (Fig. 2E), suggesting that Vav1 and HS1 function in the same pathway with respect to this function. Interestingly, Vav1 depletion decreased the rate of NK cell migration across the endothelial surface, in contrast to the increased rate caused by HS1 depletion (Fig. 2C). Here, simultaneous depletion of both Vav1 and HS1 led to an intermediate phenotype, suggesting that their molecular mechanisms have independent elements.

### Comparison to Migration in Other Cell Types

The role of HS1 in migration has been investigated in other immune cells. In neutrophils, HS1 depletion led to decreased chemotaxis to fMLP, with conventional transwell and live-cell imaging assays [22]. Neither random motility nor adhesion was affected by HS1 depletion. Tyrosine phosphorylation of three HS1 residues, 222, 378 and 397, was necessary for HS1 function in chemotaxis, based on Y to F mutations and transwell assays. The triple Y to F mutant did not rescue the chemotaxis phenotype, but the double mutant, 378 and 397, did rescue, as did all three single mutants.

In dendritic cells (DCs) from HS1 (-/-) mice, the loss of HS1 had no effect on chemotaxis in transwell assays with CXCL12 (SDF-1α) or CCL19 (MIP-3β). However, when cells were observed by video microscopy, HS1-deficient DCs displayed increased speed and decreased persistence, with cells migrating in a CCL19 gradient [16].

Finally, a recent study reveals a role for HS1 in chemokine-induced migration of human T cells [36]. SDF-1α induced rapid and transient phosphorylation of HS1 tyrosine residues 378 and 397. Phospho-HS1 interacted with Nck1, and depletion of HS1 or Nck1 impaired chemokine-induced actin polymerization and cell migration.

### Other Domains of HS1

HS1, like cortactin, has an SH3 domain and a domain that binds Arp2/3 complex [18]. Our tests of the functional importance of these domains, based on rescue of knockdown phenotypes by expression of mutants, did not reveal that either domain plays an important role in NK cell TEM. In transwell assays, the mutants provided a full level of rescue. This conclusion is limited, of course, by the assays and the cultured-cell system used in our experiments.

### Concluding Remarks

Our study provides insight into the molecular mechanisms responsible for TEM by NK cell. The findings provide information that should be helpful to understand how NK cells provide rapid responses to virus-infected cells and tumor cells. The information may be relevant to other types of cells that display TEM, such as other immune cells and tumor cells, which need to transmigrate as part of metastasis.

## Supporting Information

S1 Fig Immunoblot for levels of endogenous HS1 and expressed td-Tomato HS1 mutants in NK cells. Cells were transfected with siRNAs and expression plasmids as indicated. Anti-HS1 was used for upper panel, with anti-GAPDH below, as a loading control.
(TIF)

S2 Fig Role of HS1 in TEM by NK cells in transwell assays with depletion of HS1 by expression of shRNA. A) Number of cells in the lower chamber over time from one experiment. HS1 shRNA decreased the number, compared to control. The defect was rescued to a level higher than control by expression of shRNA-resistant HS1. Expression of wild-type HS1 produced only a small rescue effect. B) Number of cells in the lower chamber, as a percentage of the mean of the control sample value on each day. Box and whisker plots show median, 25th and 75th percentiles, and 5th and 95th percentiles. Brackets indicate statistical significance, labeled as follows: ns, not significant; one asterisk, p<0.05; two asterisks, p<0.005. The experiments were performed on three days, either in duplicate or triplicate.
(TIF)

S3 Fig Histogram of instantaneous speeds of NK cells migrating on the surface of endothelial monolayers. Instantaneous speed, plotted on the abscissa, is the distance traveled from one frame to the next in the movie, divided by the time interval. The ordinate is the number of values, on a log scale. Results are shown for NK cells depleted of HS1 and Vav1, alone and in combination. Results from three experiments on different days. In these experiments, the preparation was not treated with SDF-1α, and movies were collected immediately after NK cells settled onto the endothelial monolayer.
(TIF)

S4 Fig Histograms of persistence values for a representative experiment comparing HS1-depleted with control NK cells. Persistence, plotted on the abscissa, is defined as net displacement divided by path length. Values are calculated for the complete track for each cell in the upper panel and for sliding windows of 30, 15, 10 and 5 frames below, as indicated. Duplicate experiments, indicated as 1 and 2, were performed with control (blue, green) and HS1-depleted (red, purple) cell samples.
(TIF)

S1 Movie NK cells migrating on the surface of an endothelial cell monolayer. The NK cells were added to HDMVEC monolayers treated with SDF-1α, as described in Methods. DIC images were captured for 1 hr at 1-min intervals as a stack of three z-axis focal planes separated by 1 μm, which included the endothelial surface and the NK cells. Images were collected with a Zeiss LSM 510 confocal microscope and a 40X 1.2 NA objective
(MOV)

S2 Movie Cropped view from a movie similar to S1 Movie. (AVI)
