# Supplementary figures and images for: Role of Cortactin Homolog HS1 in Transendothelial Migration of Natural Killer Cells
Source: PLoS One. 2015 Feb 27;10(2):e0118153. doi: 10.1371/journal.pone.0118153 (PMC4344232; doi:10.1371/journal.pone.0118153)

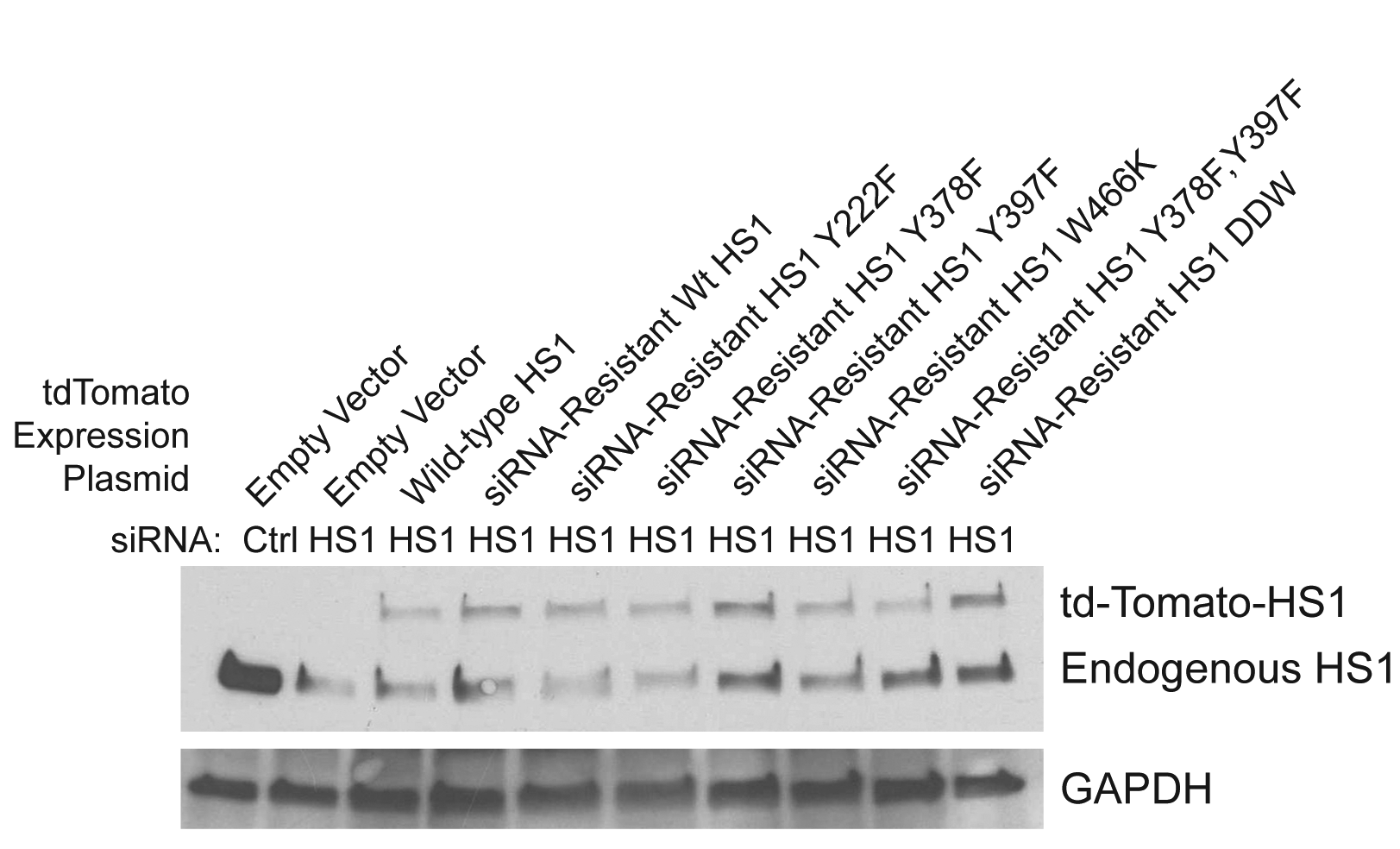

Supplement: S1 Fig — Cells were transfected with siRNAs and expression plasmids as indicated. Anti-HS1 was used for upper panel, with anti-GAPDH below, as a loading control. (TIF) [file pone.0118153.s001.tif]

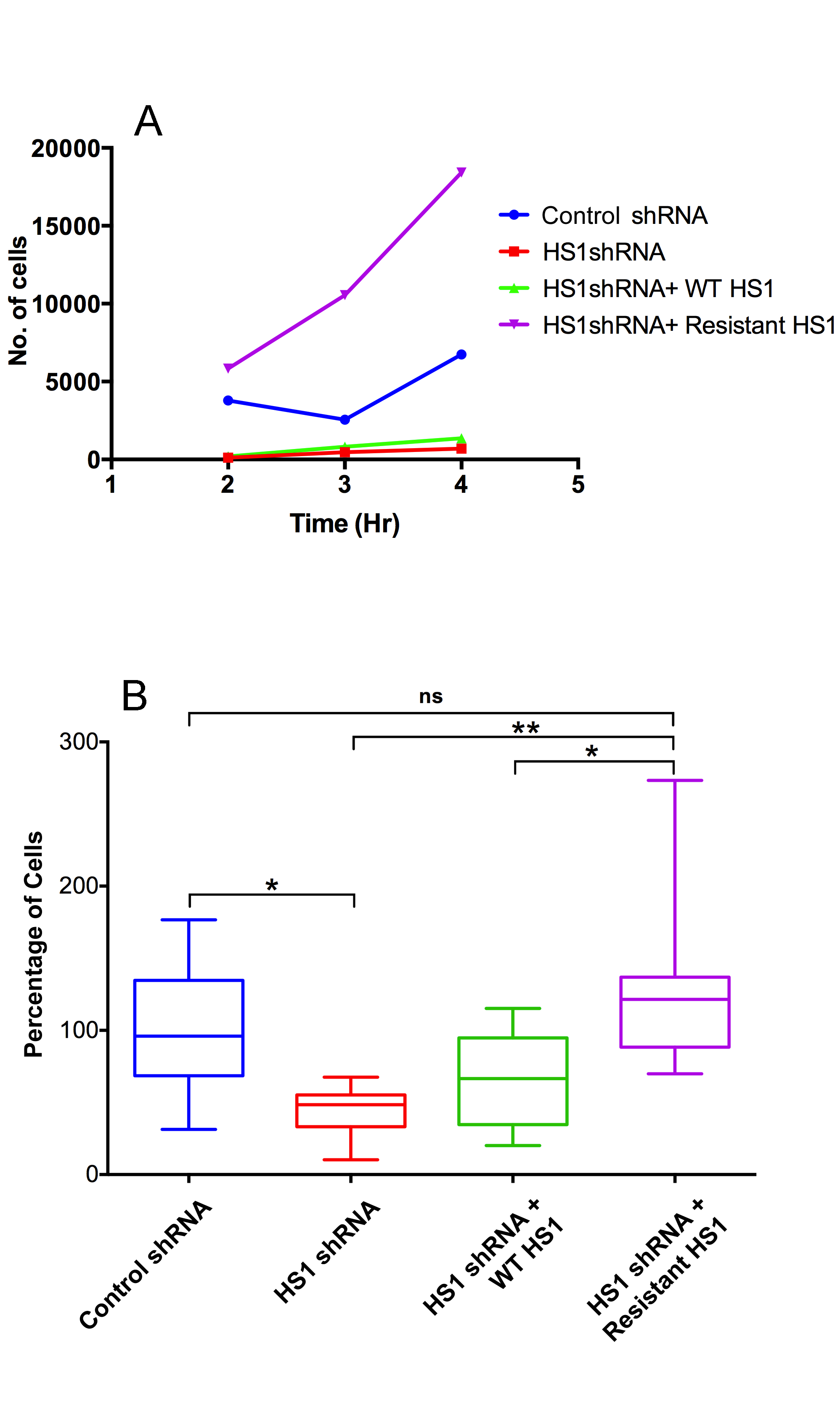

Supplement: S2 Fig — A) Number of cells in the lower chamber over time from one experiment. HS1 shRNA decreased the number, compared to control. The defect was rescued to a level higher than control by expression of shRNA-resistant HS1. Expression of wild-type HS1 produced only a small rescue effect. B) Number of cells in the lower chamber, as a percentage of the mean of the control sample value on each day. Box and whisker plots show median, 25th and 75th percentiles, and 5th and 95th percentiles. Brackets indicate statistical significance, labeled as follows: ns, not significant; one asterisk, p<0.05; two asterisks, p<0.005. The experiments were performed on three days, either in duplicate or triplicate. (TIF) [file pone.0118153.s002.tif]

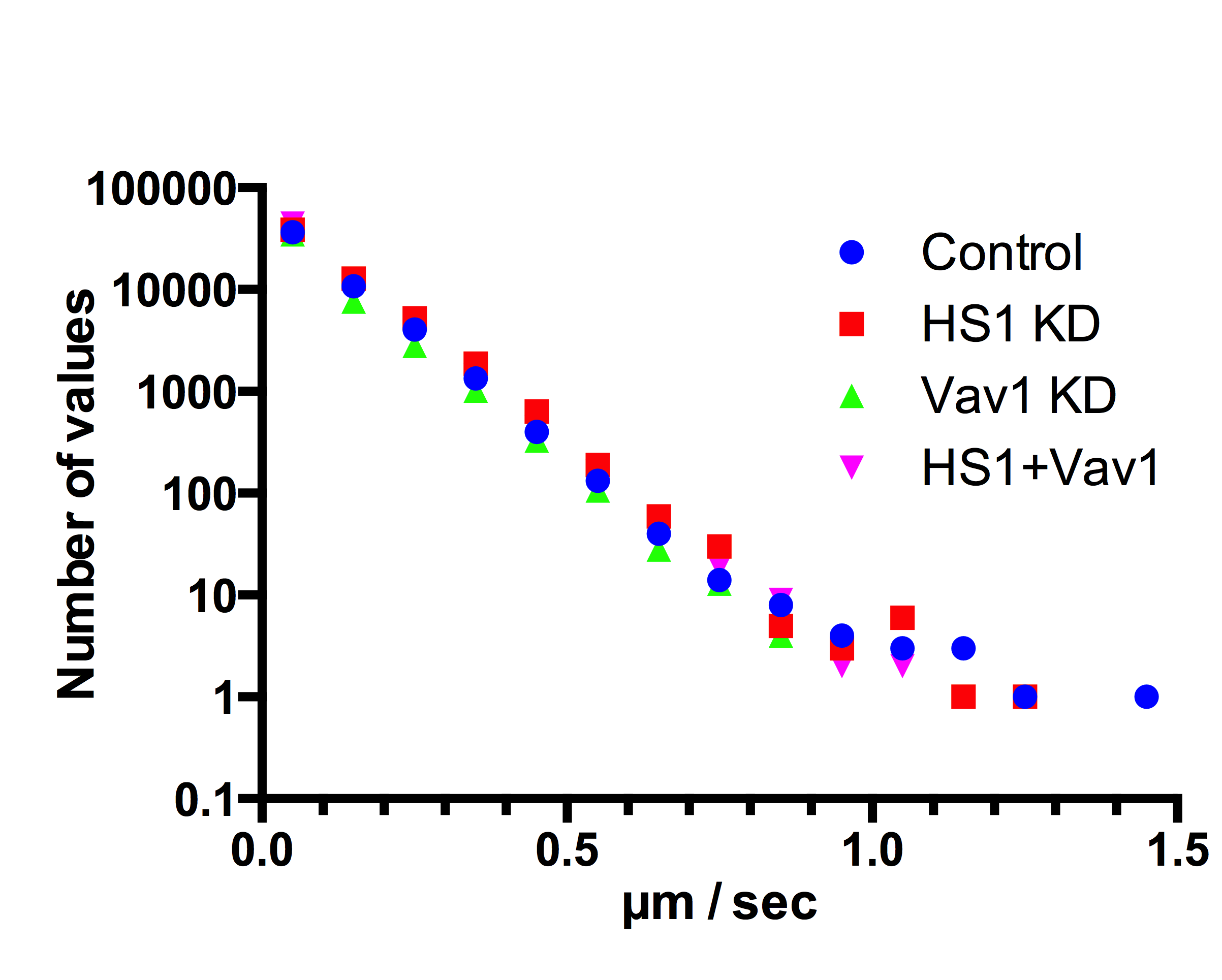

Supplement: S3 Fig — Instantaneous speed, plotted on the abscissa, is the distance traveled from one frame to the next in the movie, divided by the time interval. The ordinate is the number of values, on a log scale. Results are shown for NK cells depleted of HS1 and Vav1, alone and in combination. Results from three experiments on different days. In these experiments, the preparation was not treated with SDF-1α, and movies were collected immediately after NK cells settled onto the endothelial monolayer. (TIF) [file pone.0118153.s003.tif]

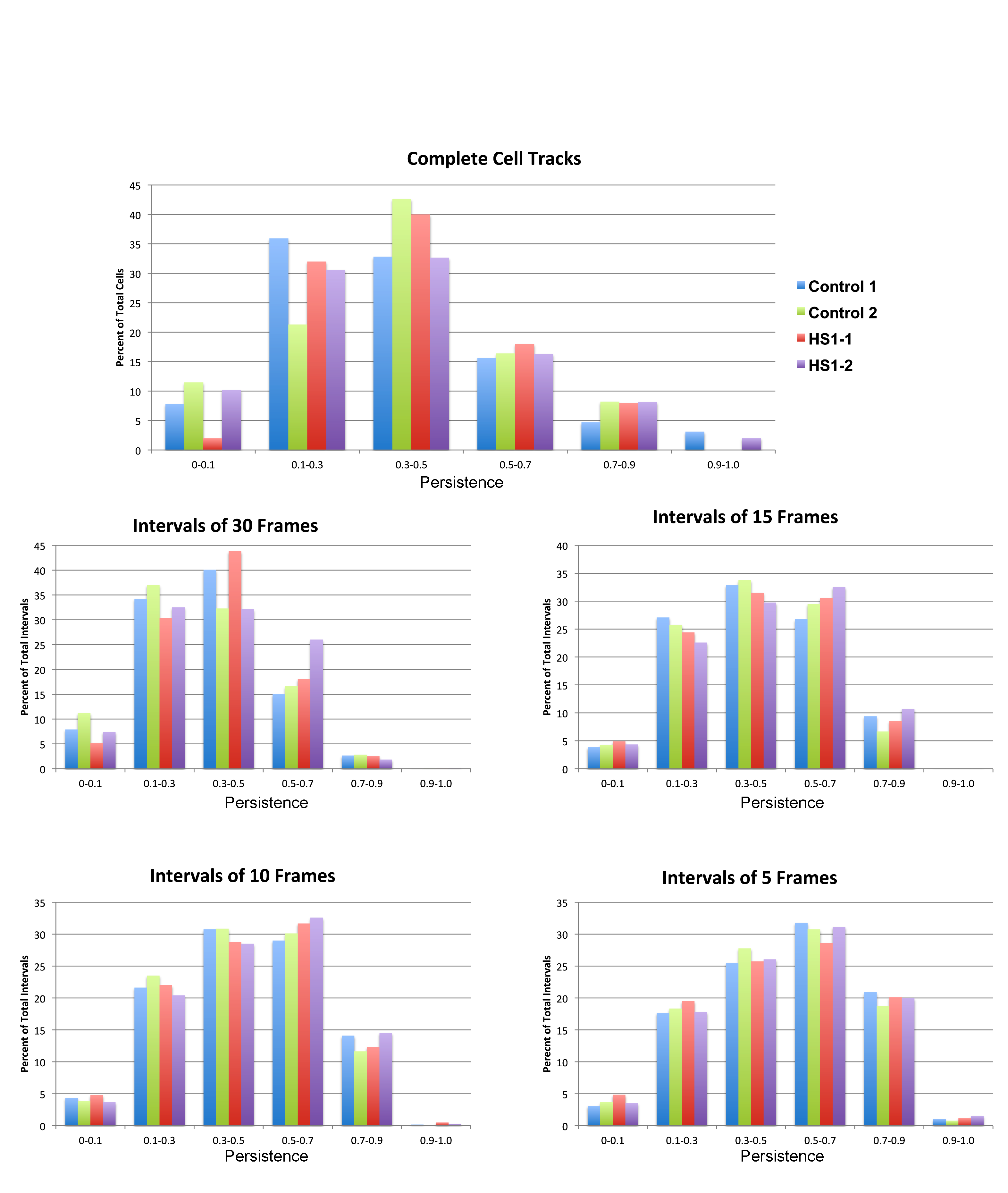

Supplement: S4 Fig — Persistence, plotted on the abscissa, is defined as net displacement divided by path length. Values are calculated for the complete track for each cell in the upper panel and for sliding windows of 30, 15, 10 and 5 frames below, as indicated. Duplicate experiments, indicated as 1 and 2, were performed with control (blue, green) and HS1-depleted (red, purple) cell samples. (TIF) [file pone.0118153.s004.tif]
